# Tests detecting biomarkers for screening of colorectal cancer: What is on the horizon?

**DOI:** 10.3205/hta000122

**Published:** 2015-06-10

**Authors:** Angaja Phalguni, Helen Seaman, Kristina Routh, Stephen Halloran, Sue Simpson

**Affiliations:** 1 NIHR Horizon Scanning Research & Intelligence Centre, School of Health and Population Sciences, University of Birmingham, United Kingdom; 2 University of Surrey, NHS Bowel Cancer Screening Southern Programme Hub, United Kingdom

**Keywords:** colorectal cancer, colorectal carcinoma, colorectal tumors, colorectal neoplasms, screening, early detection, cancer screening, cancer screening tests, early diagnosis of cancer

## Abstract

**Aim: **To identify new and emerging screening tests for colorectal cancer (CRC) that involves detection of various biomarkers like blood, DNA and RNA in samples of faeces, tissue or blood.

**Current practice: **Screening for CRC can be done by bowel visualisation techniques and tests that measure biomarkers. The Bowel Cancer Screening Programme (BCSP) in England uses a guaiac faecal occult blood test.

**Methods: **The strategy was to search available literature, identify developers and contact them for relevant information. Advice from experts was sought on potential utility and likely impact of identified technologies on the BCSP.

**Results: **Ninety-three companies and five research groups were contacted. Sixty-nine relevant tests were identified. Detailed information was available for 48 tests, of these 73% were CE marked and the remainder were considered as emerging. Forty-nine tests use immunochemical methods to detect occult blood in faeces. Eight, four and two tests detect biomarkers in a sample of blood, or exfoliated cells either shed in faeces or collected from rectal mucosa respectively. Six tests were grouped as ‘other tests’. Most of the identified tests are performed manually and give qualitative detection of biomarkers.

**Conclusion: **Variation in test performance and characteristics was observed amongst the 69 identified tests. Automated, quantitative FIT with a variable cut off are the preferred approach in the BSCP. However the units used to report FITs results do not enable comparison across products. Tests detecting biomarkers other than occult blood are more specific to neoplasms but have limited sensitivity due to the heterogeneity of cancer. Research is ongoing to identify an optimal panel of biomarkers, simplifying and automating the test, and reducing the cost.

## Introduction

**Figure 1:**
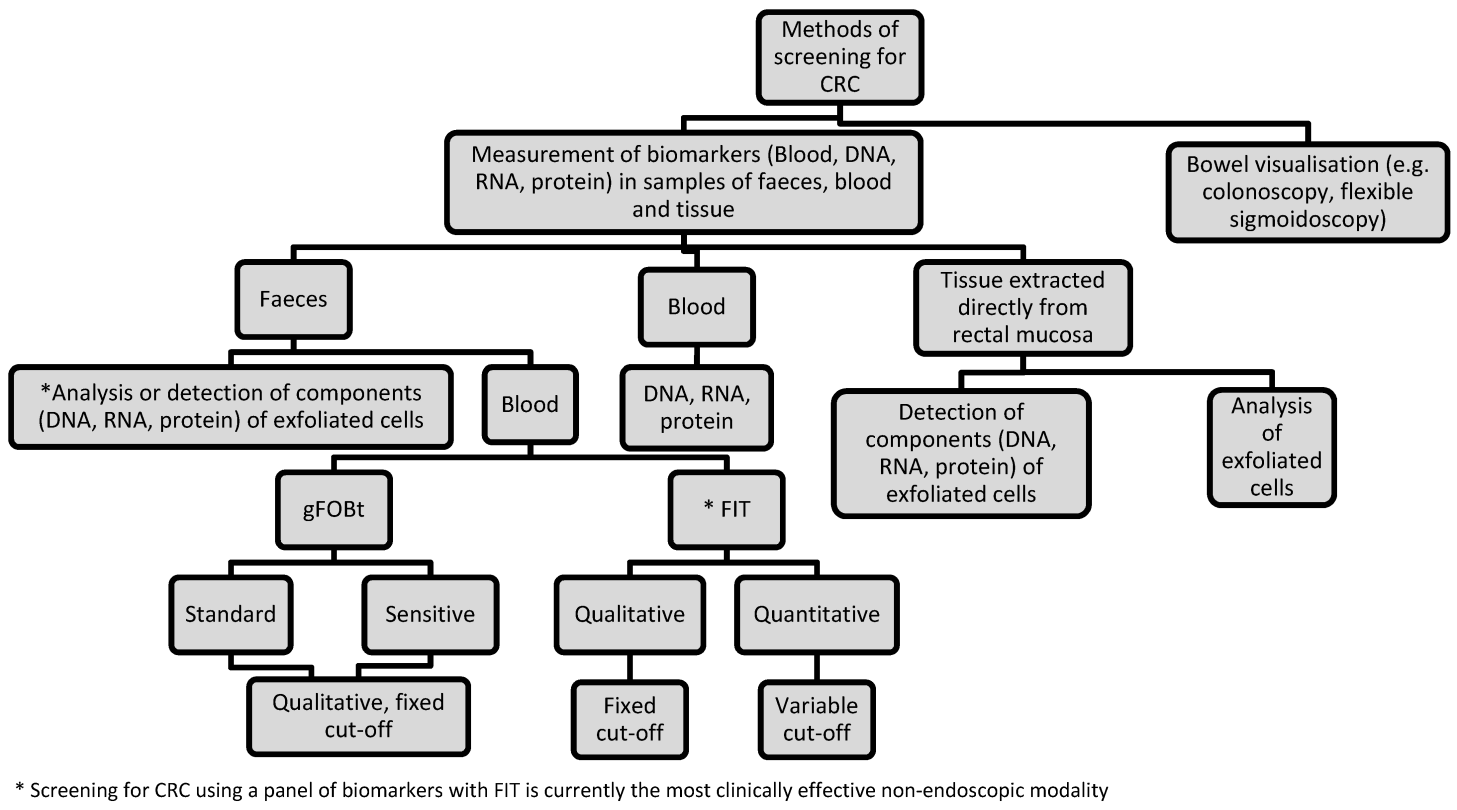
Flowchart to illustrate the various methods used for screening for CRC

Colorectal cancer (CRC) is the third most common cancer and the second leading cause of cancer death in the UK [1]. The majority of CRCs develop from small benign adenomatous polyps lining the bowel wall [2]. Progression of an adenoma into cancer takes approximately 10 years [3]. The two main methods of screening for CRC are bowel visualisation and measurement of faecal biomarkers (Figure 1 (Fig. 1)) [4]. 

Faecal occult blood tests (FOBT) are currently the most widely used screening test for CRC, based on the fact that asymptomatic colorectal neoplasia may bleed. The two main types of FOBT are guaiac FOBTs (gFOBt) and faecal immunochemical tests for haemoglobin (FIT). The gFOBt detects the pseudoperoxidase activity of the haem component of haemoglobin, whilst FIT detect the presence of globin by immunochemical reactions [5]. Other screening tests use assays that detect DNA, RNA and protein in samples of faeces, tumour tissue and blood [6], [7], [8]. These assays target single or multiple cancer-related mutations that result from disturbances of biological processes in the intestinal epithelial cells.

There is no single internationally agreed CRC screening method. Most screening programmes apply a two-step approach, which includes a non-invasive test (gFOBt or FIT) followed by a bowel visualisation technique (colonoscopy or sigmoidoscopy) for test-positive individuals, while others use colonoscopy or sigmoidoscopy as the primary screening tool. The Bowel Cancer Screening Programme (BCSP) in England offers biennial CRC screening to individuals aged 60–74 using a gFOBt followed by colonoscopy for test-positive individuals [9]. 

There is good evidence from four large randomised-controlled trials that gFOBt in population-based screening can reduce CRC mortality [10]. The gFOBt has several advantages over FIT including lower cost, good sample stability and a card-based sample collection system that enables simple and cheap mailing arrangements, although its limitations include no automated analysis, no facility to adjust the cut-off concentration for positivity, poorer participant compliance than FIT [8], [11], [12], potential interference from upper gastrointestinal (GI) bleeding, [13] and low sensitivity and specificity for adenomas and early stage CRC [11], [14]. In addition, gFOBt are susceptible to dietary interference from red meat (haem is not specifies-specific), vegetable peroxidases (false-positives) and antioxidants such as Vitamin C (false-negatives). Crude modification to the guaiac test using rehydration before analysis can increase analytical sensitivity but this makes the test more sensitive to interference from diet and unsuitable for population screening [15].

FIT are specific for human haemoglobin and so eliminate the potential for dietary interference [16]. FIT are also more specific for lower GI bleeding because globin is likely to have been degraded in faeces if it originated in the upper GI tract [16]. The superiority of FIT over gFOBts is now widely recognised and the European Quality Assurance Guideline on CRC Screening published in 2011 recommends FIT in preference to gFOBT [5], [8], [17]. Various countries have adopted FIT into their CRC screening programmes and the BCSP plans to replace gFOBt with FIT [18].

## Aim

The aim of this horizon scanning review was to provide a summary of new and emerging tests that detect biomarkers with potential for use in CRC screening.

## Method

A horizon scanning review attempts to identify and present early information on all new and emerging technologies relevant to the topic area of interest. In this review the area of interest was tests that detect blood or other biomarkers in samples of faeces, blood or tissue which had potential use for CRC screening. To ensure relevant technologies were captured, tests were included if they were emerging i.e. not yet available for use within the health care system but in development and expected to be CE-marked or launched within the next 2 years; or new i.e. tests that had been available for use in the UK for ≤5 years but may not have yet been considered for use, or were in the early phases of adoption.

Between October 2011 and March 2012, potential tests were identified using recognised horizon scanning methods [19]. These included a combination of internet searches, expert suggestions and direct contact with companies (Table 1 (Tab. 1)). For technologies in development or in the early phases of adoption, it is common for there to be a lack of publicly accessible information and scientific data available. This is particularly true for non-pharmaceutical technologies such as diagnostic tests. Therefore further information about the initial long-list of individual tests identified was requested from developers. This involved contacting companies or research institutions directly using a standard proforma developed by the NIHR Horizon Scanning Research & Intelligence Centre (HSRIC). Information requested included test description (e.g. name, synonyms, intended indication; test method; biomarkers detected etc.); details on the usability of the test (e.g. details of specimen collection, dietary restrictions, transportation requirements etc.); the test process, analysis and interpretation of results; stage of development; cost and data on test accuracy. If no contact with the company could be established, a focused internet search was carried out for individual technologies to obtain any information available. 

When information had been collated for the initial list of potential screening tests, they were categorised as either ‘emerging’ or ‘new’ based on their development timeframe. Tests that were found to be established were excluded from the final list. An expert advisory group (Table 1 (Tab. 1)) were sent the final list and invited to provide informed opinion on the potential utility, advantages and/or disadvantages of tests. The resulting output was a descriptive list of new and emerging tests detecting biomarkers that have potential for use in CRC screening. No analysis of evidence data obtained during the identification stages was carried out. The descriptive list can be used by those commissioning and planning further research (both primary and secondary), policy makers (in this case national screening committees), health care funders and health care practitioners.

## Results

We identified 145 tests involving detection of biomarkers that had potential for use in CRC screening; these were provided by 93 companies and five academic research groups. Sixty-one (62%) of the companies were able to provide the information requested and 69 (48%) tests met the review inclusion criteria. The remaining 76 tests (52%) were excluded, because they did not meet the inclusion criteria for a new or emerging test, or the test was a duplicate of another that had been identified. We obtained details on the development status of 48 tests. Fifteen tests (31%) were CE marked and available for clinical use in the NHS, eight (17%) were CE marked but not yet available for clinical use in the NHS, 12 (25%) were CE marked but information regarding clinical use in the NHS was not available, and 13 (27%) were considered as emerging. 

**Figure 2:**
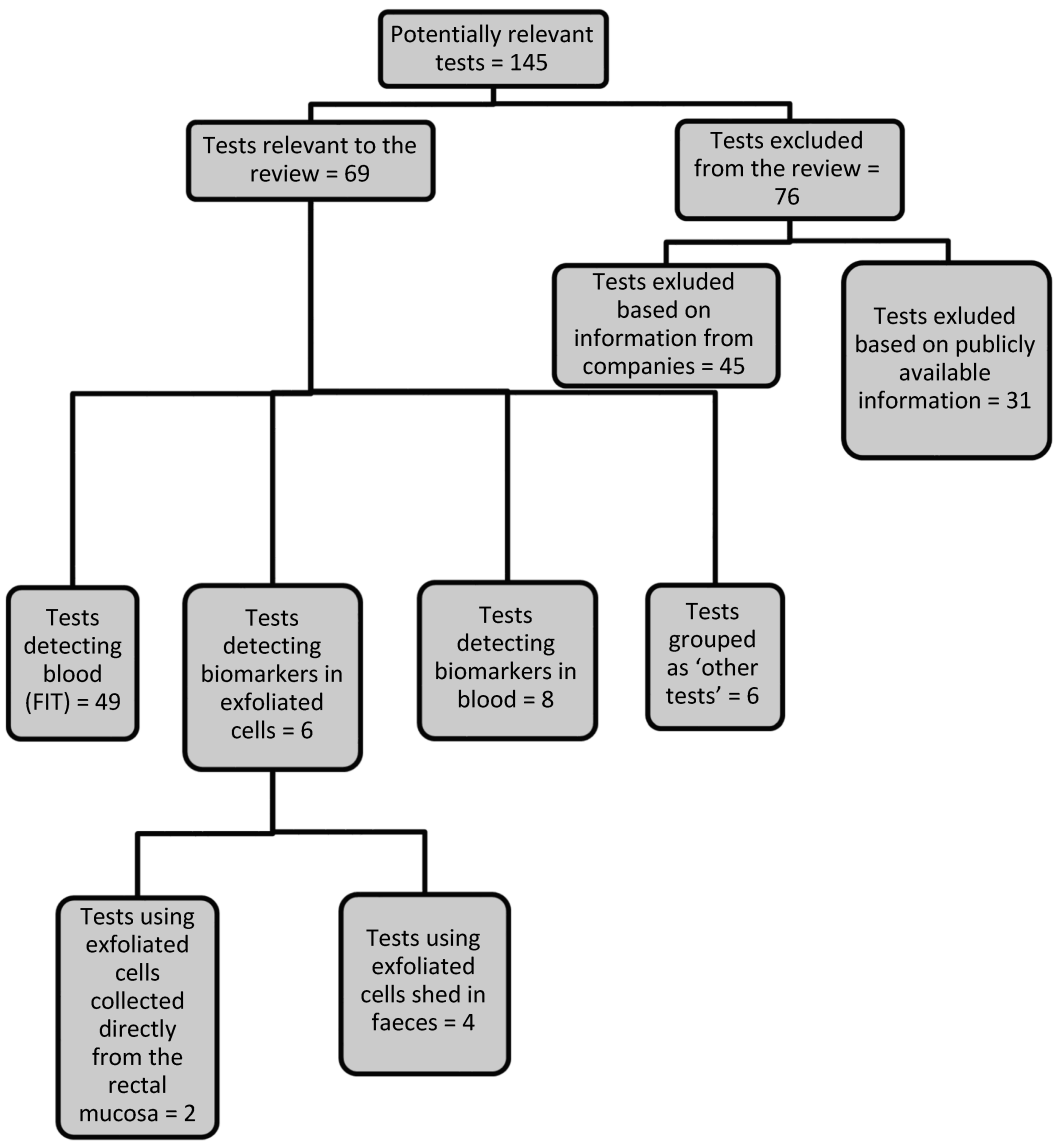
Flowchart to illustrate key findings of the review

Figure 2 (Fig. 2) summarises the 69 tests included in the review and lists the analytical methods used to detect and measure the biomarkers. The majority of tests (49; 71%), including four emerging tests, use an immunochemical method to detect blood in faeces. Eight (11.6%), including six emerging tests, detect other biomarkers in blood; four (5.8%) detect biomarkers in exfoliated cells shed in the faeces, and two (2.9%) emerging tests use exfoliated cells collected directly from the rectal mucosa. Six (8.7%) tests, including one emerging test, were grouped as ‘other tests’ (as the test method used and/or the substance detected by these tests did not allow for them to be grouped under the test categories mentioned above). Only eight of the 13 tests considered as ‘emerging’ could be considered as innovative, using a novel method or detecting biomarkers different from existing marketed tests. Of these, five tests (products 56, 57, 58, 59 and 60) detected biomarkers such as methylated septin 9, a panel of DNA biomarkers, antibodies against p53 proteins or circulating tumour cells in a sample of blood; two tests (products 61 and 62) use exfoliated cells collected directly from rectal mucosa to measure the concentration of DNA or analyse the alterations in the nanoscale architecture of colonocytes by using a partial wave spectroscopic microscopy instrument; and one test (product 65) detects the amount of cancer-associated carbohydrate in rectal mucus.

Table 2 (Tab. 2) provides details of the tests identified (confidential data prevented inclusion of one FIT test). The majority of FIT (94%) identify blood in faeces by detecting globin, some (6%) detect haemo/haptoglobin complexes. Three tests (products 8, 26 and 45) detect globin and other proteins such as transferrin or ferritin. Based on available information on 45 FIT, 30 (67%) use immunochromatography (enzyme immunoassay in lateral flow systems) to detect blood in faeces (qualitative devices). Other immunochemical test methods include colloidal gold agglutination (n=1), reverse passive haem-agglutination (n=1), magnetic particle agglutination (n=1), immunoturbidimetry (n=6), and enzyme immunoassay/ELISA (n=6). Eleven FIT (22%) provide a quantitative measurement of faecal human haemoglobin concentration and analysis can be automated. Thirty-three FIT (67%) provide qualitative detection of human haemoglobin, of which only two can be automated (products 22 and 27); the others require visual interpretation of test results. 

The main method of specimen collection for tests that detect biomarkers in faeces involves collection of a single faecal sample into liquid buffer contained in specialised collection tubes. Only three FIT (products 13, 21 and 44) devices use dry cards for collection of the faecal specimen. None of the tests included the need for any dietary restrictions. The cost of tests that detect blood in faeces is generally considerably lower than tests that detect other biomarkers in samples of faeces, exfoliated cells or blood. 

Information on cut-off concentrations (for FIT), sample stability and clinical sensitivity and specificity of the tests for CRC or adenomas was available for some tests. However, the importance of consistency in the way this information is reported has only recently been recognised [20], [21] which can make simple comparison across products both inaccurate and misleading.

## Discussion

A large number of potential markers for CRC screening was identified, the majority of which are already available for clinical use in the UK and relatively few of which use a novel approach. Most new and emerging identified tests were FIT. The 49 FIT that were identified varied in their reported test performance (clinical sensitivity and specificity for CRC and adenomas) and test characteristics (substance detected, test method, method of sample collection, cut-off and type of detection). 

A non-invasive, inexpensive, clinically validated test that is acceptable to users and facilitates automated high-throughput testing would be ideal for a population-based screening programme. FIT are superior to gFOBts as a screening test for CRC, primarily because of greater clinical sensitivity, specificity and screening compliance [11], [14]. Improved compliance may be due to the need for a single sample and an easier stool collection method. The use of a liquid buffer to help preserve haemoglobin in FIT presents difficulties and expense for safely mailing the test kits to and from subjects [22]. Whilst new buffers are appearing on the market, the stability of haemoglobin in FIT sample collection tubes is generally poorer than that of haemoglobin applied to a gFOBt [23].

Several factors might influence the stability of haemoglobin in a buffer solution including temperature [24] and sample return time [25]. To slow down the degradation of haemoglobin, in most new and emerging FIT, the sample is added to a preservative buffer and the advice is to store at temperatures lower than room temperature. Information on sample stability was received from some companies, but as the definition of stability has not yet been standardised it makes company claims difficult to compare.

Both qualitative and quantitative FIT were identified. Qualitative tests are not appropriate for a large screening programme since the concentration at which tests become positive is set by the manufacturer and demand for colonoscopy is effectively set by the manufacturer [23]. Qualitative tests, which use laminar flow immunochromatography, require subjective visual assessment of results and make quality assurance difficult. Consistency in interpretation of test results could be improved by designating a centralised location for analysis of all tests or by using tests with automated reading [26]. Analysis of only two of the qualitative FIT identified (products 22 and 27) can be automated.

Quantitative FIT generally use automated analysis to determine the faecal haemoglobin concentration, which allows high throughput testing, improves reproducibility and removes inter-observer variation in interpretation of test results [5]. A further benefit of quantitative FIT is that the cut-off concentration can be adjusted according to colonoscopy capacity and the intended detection rate in the screened population [4]. Since the amount of faecal haemoglobin is likely to be higher in patients with CRC than in patients with advanced adenomas, a difference in the cut-off will affect primarily the detection of advanced adenomas [14], [27], [28], [29], [30]. The relationship between the concentration of haemoglobin in faeces and the risk of cancer or advanced adenoma has yet to be fully exploited with quantitative tests. These tests will enable more effective screening algorithms to be derived which combine haemoglobin concentration with other parameters such as screening history, recent endoscopy, and the age and sex of the subject [31]. Current FIT measure the concentration of haemoglobin in the buffer (*e.g*. ng haemoglobin/mL of buffer solution). The numeric cut-off concentration, if quoted in mass of haemoglobin in a device buffer, will be directly related to the design of a FIT, the volume of the buffer solution it holds and the mass of sample it collects [20], [21]. The cut-off is therefore unhelpful when comparing performance of one FIT with another. To perform such comparisons it is recommended that FIT results be reported as concentration of haemoglobin in the faecal sample (µg haemoglobin/g faeces) [20], [21].

Both gFOBts and FIT require faecal sampling and this is likely to be a barrier to high compliance [32]. Three tests (one FIT [product 21] and two ‘other tests’ [products 63 and 66]) use methods that minimise or eliminate the need for faecal handling; the FIT uses a long-handled brush collection system and the others use a test tissue that is dropped into the toilet bowl after a bowel movement. However, both these products are vulnerable to potential interference from disinfectants and other cleaning products added to toilet water.

To be able to choose the most clinically-effective FIT testing system, it is essential that results from different systems can be compared and that critical steps in the analytical and reporting process are standardised. FIT currently use different analytical materials, report results unique to the individual device and have no consensus method of measuring and reporting sample collection mass or test stability. To aid the difficult process of selecting a test kit and cut-off concentration appropriate to individual screening programmes the World Endoscopy Organization formed an Expert Working Group ‘FIT for Screening’ in 2011 to identify and promote standardisation of FIT [16], [20], [21], [33], [34], [35].

The clinical performance of gFOBts and FIT is affected by lesions that bleed intermittently (sensitivity) and by non-specific bleeding from lesions other than CRC and adenomas (specificity). Fifteen new and emerging tests that detect biomarkers other than blood in faeces were identified. Like FIT, these varied in test performance and characteristics, although unlike FIT, the biomarkers detected by these tests will be more consistently present and are more specific to neoplasms [36]. Nevertheless, the decision to use them in screening might bring additional difficulties. Tests that detect biomarkers in exfoliated cells shed in faeces still require faecal handling and that is linked with poor compliance and poor quality of biomarker due to faecal contamination could decrease test sensitivity. Also, human DNA accounts for roughly 0.01% of faecal DNA and the remaining DNA is either from microflora or diet [37]. The stability and isolation procedures of human DNA from stool are affected by the abundance of bacteria and cytolytic substances in the faeces [38]. Quality of biomarker from the faeces can be improved with a new purification method to extract high quality biomarkers from a larger faecal sample than that used for gFOBt or FIT [39]. Screening tests that use exfoliated cells collected directly from rectal mucosa are unlikely to prove feasible for a screening programme because of the resource cost (*e.g.* healthcare staff time) associated with performing a digital rectal examination to collect the sample. In addition, the invasive nature of this procedure and the need to attend a clinic appointment are likely to be deterrents.

A number of new and emerging tests detecting biomarkers in blood were identified that may have benefits over stool testing. Firstly, there are few microflora that can decrease the quality of the biomarker [40] and secondly a test in blood might improve screening compliance since a single blood test might prove more convenient and acceptable to the screening participants [40]. If a blood sample is being taken for other clinical reasons, perhaps as a general ‘wellness assessment’, this might prove attractive, particularly in countries that have well-developed disease prevention programmes. A blood sample carries associated healthcare costs and might be more relevant to the proportion of screening population that is unwilling or unable to collect faecal samples. 

Biomarkers such as methylated DNA are present in very small amounts in blood which makes them less attractive for detection of early stage cancers and adenomas [6]. No single biomarker has yet yielded perfect sensitivity to CRC and advanced adenomas due to the heterogeneity of the tumours [37]. Use of a panel of biomarkers will increase sensitivity while potentially maintaining specificity [7]. Three tests (products 54, 56 and 60) identified in this study detect a panel of biomarkers but this further increases the complexity and the cost of the test. Lack of large scale population-based validation studies and the uncertainty about the frequency of administration of these tests has further limited their clinical utility [37], [41]. Since the review was conducted, US investigators have reported on the performance of FIT versus a panel of stool DNA markers for CRC plus FIT and concluded that the panel of CRC markers showed higher single-application sensitivity than FIT alone for both CRC and advanced precancerous lesions (including sessile serrated adenomas), although with lower specificity [7]. The panel test requires a complete stool sample, which introduces extra cost and impracticality to an already expensive testing procedure. Research will continue to identify either a sensitive single biomarker or a panel of biomarkers that simplify and automate the testing and do so at an affordable cost [8].

## Study limitations

The majority of the tests identified were either developed by, or have the support of, a major *in vitro* diagnostic manufacturer; some of the more sensitive information was frequently regarded as ‘commercial in confidence’. The nature of the *in vitro* diagnostic industry has also presented a number of challenges since tests are often developed by individuals, universities, or small start-up companies and then acquired, or merged with, larger companies as the commercial opportunities are realised. This process is confounded by product and company names changes which may account for the lack of response to our requests for information from some companies. 

The methods used to identify technologies and obtain further information about those technologies can mean that the data obtained is not always current, can be accurate or can be subject to bias. These limitations are minimised by using a variety of identification sources and involving subject experts.

Identification of the tests took place during October 2011 to March 2012 resulting in some of the information presented being out of date. The nature of horizon scanning is that information identified on technologies that are in development is continually changing. However, the information presented provides a snap-shot of this rapidly changing field. The importance of this information being to alert policy makers, commissioners, research funders and evaluators to the developments in this field and to raise awareness of the range and complexities of the emerging tests. This information feeds into planning and is not generally available elsewhere other than through expensive market reports.

## Conclusion

There is a recognised need for a screening test for colorectal cancer that is non-invasive, inexpensive and acceptable. The test should have sufficient clinical sensitivity to produce cost effective reductions in the incidence and mortality of CRC, and a clinical specificity to minimise unnecessary colonoscopies, patient anxiety and cost to the health care system. FIT were the most common new screening tests identified in this study which indicates a renewed interest in faecal blood testing and the potential for further product enhancement. Automated quantitative FIT with a variable cut-off are currently the preferred test for CRC screening programmes but a test reliant upon the detection of blood in faeces has inherent limitations. Advanced adenomas and early cancers should be the target lesions of a screening programme designed to reduce both incidence and mortality of CRC. These early lesions may bleed lightly and intermittently, if not at all, and will therefore elude FIT detection. The holy grail is a highly sensitive test that is specific for CRC. Emerging tests use biomarkers other than blood, for example DNA and specific proteins both in blood and in faeces, or a panel of biomarkers with FIT. To facilitate the decision on the adoption of various tests identified in this review, and to ensure the NHS secures the most cost and clinically-effective testing approach, it is essential that data on the clinical validation of new and emerging tests in the screening population is made available.

The search for a better test for CRC continues and whilst new products appear on the market, they will have to demonstrate that they are both more clinically effective and affordable for population-based screening.

## Notes

### Competing interests

The authors declare that they have no competing interests.

### Funding 

The study was undertaken as part of the research programme of the NIHR Horizon Scanning Research & Intelligence Centre (NIHR HSRIC). The NIHR HSRIC is funded by the National Institute for Health Research (NIHR). This article presents independent research funded by the NIHR.

## Figures and Tables

**Table 1:**
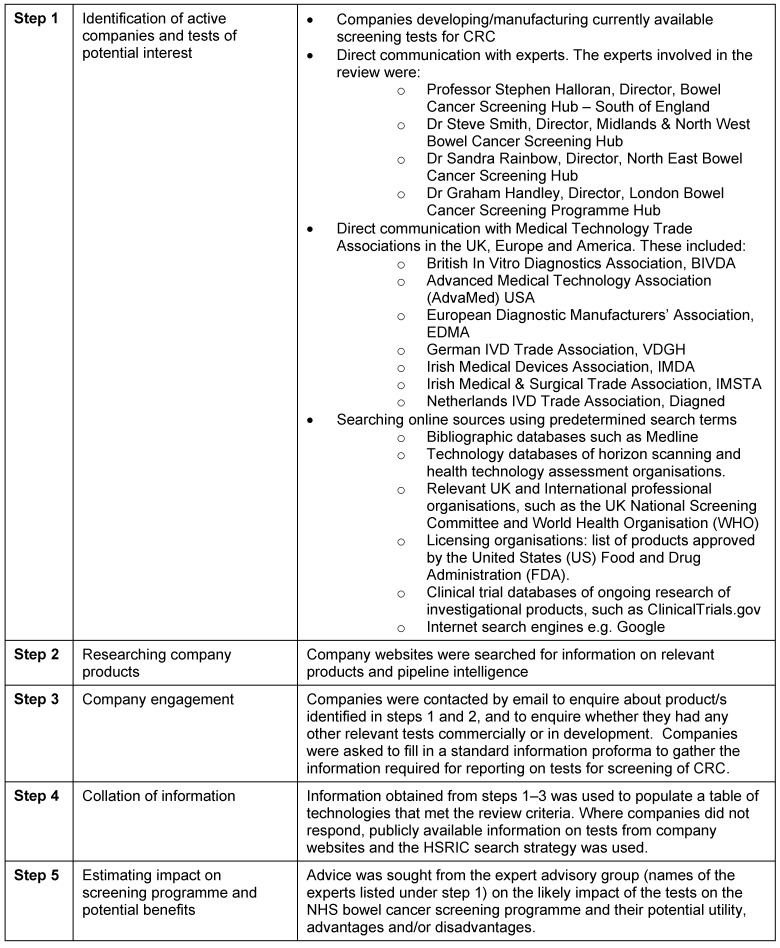
Steps involved in conducting the horizon scanning review

**Table 2:**
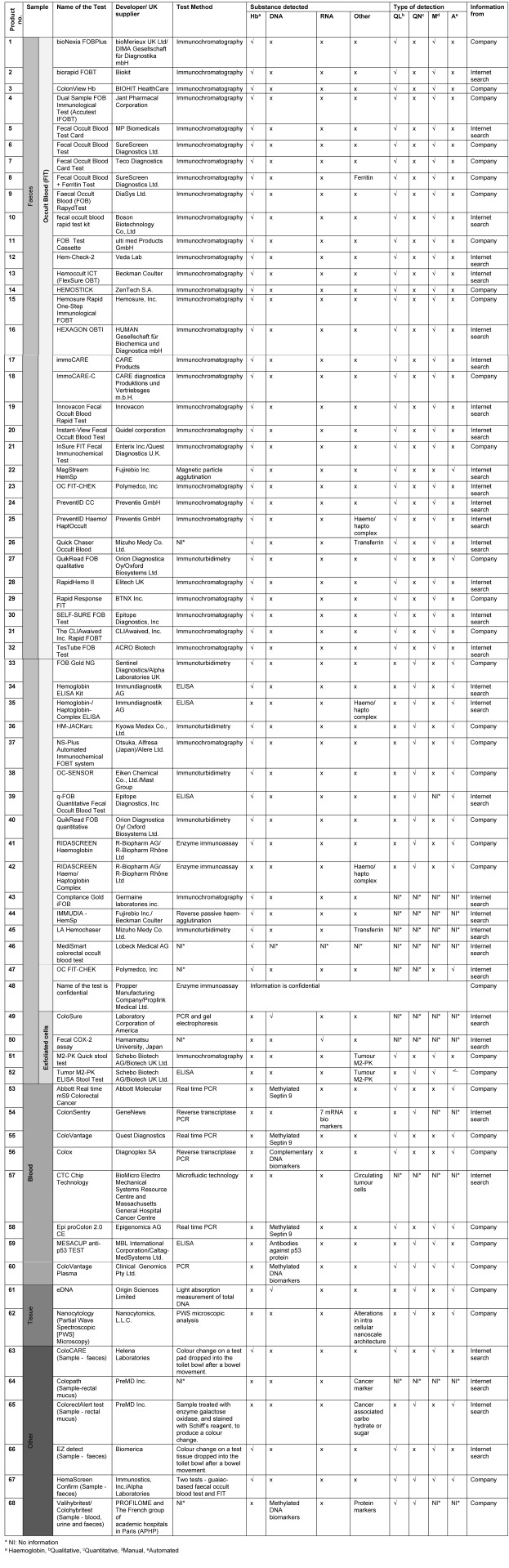
Characteristics of tests for screening of CRC included in the study
